# From a Molecule to a Drug: Chemical Features Enhancing Pharmacological Potential

**DOI:** 10.3390/molecules27134144

**Published:** 2022-06-28

**Authors:** Giovanni Ribaudo, Laura Orian

**Affiliations:** 1 Dipartimento di Medicina Molecolare e Traslazionale, Università degli Studi di Brescia, Viale Europa 11, 25123 Brescia, Italy; giovanni.ribaudo@unibs.it; 2 Dipartimento di Scienze Chimiche, Università degli Studi di Padova, Via Marzolo 1, 35131 Padova, Italy

Health is a fundamental human right and is a global goal to which extensive research effort is devoted in all fields. Chemistry plays a key role in understanding the mechanisms ruling health and disease conditions at the molecular level, as well as in discovering substances with pharmacological potential which can restore health status or mitigate pathology-related damage. One of the major challenges is to understand, rationalize, and control those molecular features which are crucial for a specific drug action. This problem is rooted in the well-known chemical ambition of establishing structure–activity relationships of general validity, although other relevant aspects must be considered, such as solubility, targeting efficiency, and toxicity.

Stitching to the first essential aspect, we assist the continuous evolution of the chemical design approach, which was mainly based on the expensive ‘trial and error’ method only few decades ago. It is commonly accepted that the trials can be efficiently delegated to computers. Machine-assisted drug design has gained importance with the implementation of different methodologies, ranging from quantum chemistry to classic and continuum approaches, and, more recently, with the application of artificial intelligence algorithms. Despite the fact that there is plenty of room for improvement, large-scale screenings, protein–ligand and protein–protein docking, simulations, and molecular- and multi-scale mechanistic studies play an important role in research progress and receive a large consensus in health sciences.

When we conceived this Special Issue, it became apparent for us to choose a topic and title which reflect our different background in medicinal and theoretical computational chemistry and is close to our joint collaboration. Combining our complementary expertise, we recently developed a project repurposing or better redesigning a popular antidepressant drug molecule, i.e., fluoxetine, which is better known by its commercial name, Prozac. We designed in silico a series of selenoderivatives of fluoxetine and assessed their enhanced antioxidant capacity through chemical and computational protocols [1,2], and, finally, we demonstrated in vivo that selenofluoxetine maintains its SSRI antidepressant action [3]. These outcomes paved the route to our contribution on this Special Issue, in which we report on a new ability of these selenofluoxetine derivatives, i.e., a novel strategy to selectively release bioactive molecules within a selenoxide elimination-triggered enamine hydrolysis [4].

The Special Issue collected contributions from researchers all over the world, demonstrating the flourishing interest of the international scientific community towards the abovementioned aims and scopes. Amalia Stefaniu and colleagues reported a computer-aided screening of benzoic acid derivatives and semisynthetic alkyl gallates against SARS-CoV-2 main protease [5]. Furthermore, the paper from Amin Osman Elzupir focuses on the SARS-CoV-2 outbreak, but a different mechanism was considered, as the author presented an in silico evaluation of pyrimidonic pharmaceuticals against papain-like protease [6]. In their review article, Sebastián A. Cuesta and Lorena Menes provided an overview on the evolution of analgesic and anti-inflammatory drugs, including theories on novel mechanisms of action [7]. Everaldo F. Krake and Wolfgang Baumann used NMR to investigate the reactivity of clopidogrel towards reactive halogen species [8]. Giuseppe Zagotto and Marco Bortoli provided a perspective on the evolution of medicinal chemistry, which nowadays faces novel challenges in the context of precision medicine and advanced drug delivery [9]. This aspect was also approached by Karolina Wanat and Elżbieta Brzezińska, who studied the effects of protein binding on drug bioavailability by means of statistical methods related to molecular and chromatographic descriptors [10], and by Tsun-Thai Chai and colleagues, who predicted pharmacokinetic and pharmacodynamic properties of seafood paramyosins peptides though computational tools [11]. Hoang Thai Ha and colleagues presented a comprehensive study on the extraction, characterization, and evaluation of antioxidant activity of carrageenan from *Eucheuma gelatinae* [12]. Daniel Muñoz-Reyes and colleagues described a novel application for a known molecule, investigating the role of quercetin 3-*O*-glucuronide against cisplatin cytotoxicity in renal tubular cells [13]. Ivan Yu Torshin and colleagues provided novel insights on the use of a known therapeutic agent, as they reported their study on lithium salts with reduced toxicity as neuroprotective agents [14]. In the context of neuroprotection, Etimad Huwait, Dalal A. Al-Saedi, and Zeenat Mirza presented a combined in silico and in vitro study assessing the potential of fucoidan against atherosclerosis [15]. In their analytical chemistry-oriented contribution, Elena Alba Álvaro-Alonso focused their study on the investigation of physicochemical and microbiological of oral solutions of methadone in different storage conditions [16].

As a conclusive note as Guest Editors, we would like to sincerely thank all the authors for choosing our Special Issue to share the results of their research work, as well as the reviewers and the assistant editors for their valuable support.
